# Effect of Ionic Liquids on the Structure of Ionomer Inks

**DOI:** 10.1021/acs.macromol.5c00698

**Published:** 2025-07-28

**Authors:** Tyler B. Martin, Kimber Stamm Masias

**Affiliations:** † Materials Science and Engineering Division, 10833 National Institute of Standards and Technology, Gaithersburg, Maryland 20899, United States; ‡ Toyota Motor North America R&D, Ann Arbor, Michigan 48105, United States

## Abstract

This study investigates the impact of ionic liquids (ILs) on the structure and performance of catalyst inks used in proton exchange membrane fuel cells (PEMFCs). Using contrast variation small-angle neutron scattering (CV-SANS), we elucidate the structural interactions between ionomers, Pt/C catalyst particles, and ILs. The analysis reveals that IL composition significantly influences the arrangement of ionomers and their interactions with other ink components. In particular, one of the ILs ([BMIM]­[C4]) exhibited stronger associations with both ionomer and catalyst particles, resulting in reduced availability of platinum catalytic sites and lower PEMFC performance. These findings demonstrate the structural origins of electrochemical performance variations, providing insights into the design of improved ionic liquid formulations for enhanced fuel cell efficiency.

## Graphical abstract

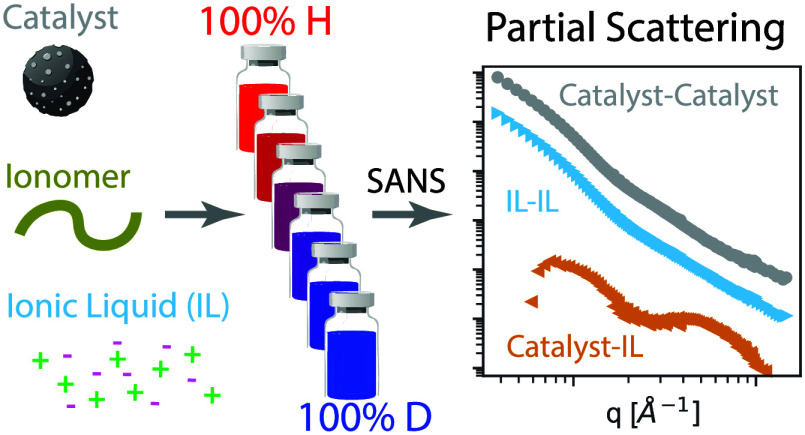

## Introduction

Proton exchange membrane fuel cells (PEMFC) convert chemical energy from the reaction of H_2_ and O_2_ into electricity, presenting a low emission route to a carbon neutral energy source. A major component of a PEMFC is the membrane electrode assembly (MEA) which consists of gas diffusion layers, catalyst layers, and the polymer electrolyte membrane. While reducing the unit cost by replacing the platinum (Pt) catalyst is a large and ongoing focus of researchers, other challenges persist. Efficiency of PEMFCs is hampered by energy losses at the cathode during the oxygen reduction reaction,^1^ opening the door to investigation of non-Pt catalysts,^2^ shape-controlled catalysts,^3^ alternative ionomers for proton transport,^4^ and improving the triple phase boundary between carbon, Pt, and ionomer.

Searching for ever-better performance, researchers have investigated the inclusion of ionic liquids (ILs) in the ionomer complex with the aim of improving the efficiency of O_2_ diffusion through the polymer by modifying the ionomer-Pt catalyst interface.^5–8^ Several investigations have shown the inclusion of ILs improves the O_2_ transport kinetics as well as the accessibility of Pt catalyst sites. Several electrochemical origins for the change in performance can be proposed, including changes in hydrophobicity that impact proton transport or variations in the O_2_ transport kinetics. Structural origins for the performance improvement, such as changes in the association between the ionomer side chain thiol groups and the Pt catalyst, are also plausible. These explanations have been considered in the dried catalyst layer of the MEA but the impact of including the ionic liquid into the catalyst ink used to create the MEA have not been explored.

In order to fabricate a functional MEA, all components of the MEA are mixed and suspended in a catalyst ink using water and alcohol as solvents. The structure and eventual function of the MEA is directly impacted by the properties of the ink, including the coating of the ionomer onto the carbon-supported Pt catalyst (Pt/C) and the subsequent aggregation of the catalyst particles themselves. Identifying the impact of the ILs on the ordering and aggregation of the ink components will allow a better understanding of the structural rearrangements of the realized MEA that modify its electrochemical performance.

Measuring the arrangement of the catalyst ink components using traditional techniques, such as small-angle scattering, is challenging. The contrast and fractal assembly of the carbon and Pt components of the ink dominate the signals of both small-angle X-ray and neutron scattering (SAXS and SANS, respectively). Even attempting to leverage simple contrast matching techniques in SANS fails due to the strong scattering and neutron SLD of the carbon and Pt.

To overcome this, previous studies have used contrast variation SANS (CV-SANS) to study the catalyst layer inks (without ionic liquids).^9–12^ Using CV-SANS, these authors were able to identify the structural arrangement of the ionomers in their inks, despite raw SANS intensity being dominated by the Pt/C. The CV-SANS approach takes the SANS pattern of a material measured at several contrast conditions and decomposes the scattering into individual component scattering functions. This allows for the understanding of not only the overall solution structure of the ink, but also the relative arrangement of the individual components (e.g., IL, ionomer, Pt/C).

In this study, we use CV-SANS to determine the impact of multiple ionic liquids on the structure of a liquid phase catalyst ink. We show that traditional, analytical SANS analysis of the inks produces ambiguous results. In particular, attributing the trends in length scales and fractal dimension to specific components of the ink is challenging, even when comparing the values at multiple contrast conditions. Using the CV-SANS technique, we elucidate both the structure and cross-interactions between the Pt/C particles, ionomers, and ionic liquids. We find that one of the ILs (IL1, defined in Table 1), shows much stronger association with both the ionomer and Pt/C (particle) components of the ink than the other ILs considered. In contrast, inks containing IL2 and IL3 show weaker associations between the ILs and the other ink components, but stronger ionomer-particle associations compared to the IL1 system. By comparing these structural observations of the inks with performance measurements of a realized PEMFC, we show that the increased IL1-iononmer and IL1-Pt/C interactions are correlated with poorer measured performance.

**Table 1:** Summary of Different Systems Studied^a^

label	carbon	platinum	[BMIM][C4] (IL1)	[MTBD][C4] (IL2)	[MTBD][TFSI] (IL3)
C	x				
PtC	x	x			
PtCIL1	x	x	x		
CIL1	x		x		
PtCIL2	x	x		x	
PtCIL3	x	x			x

a All systems contain ionomer and a suspending solvent (ethanol + H_2_O), as described in the Sample Preparation section.

## Methods

### Sample Preparation

Figure 1 is a schematic depiction of the inks considered in this study, while Table 1 shows the specific mixtures considered along with their identifying labels. The two IL cations considered were 1-butyl-3-methylimidazolium ([BMIM]) and 7-methyl-1,5,7-triazabicyclo[4.4.0]­dec-5-ene ([MTBD]) and the anions 1,1,2,2,3,3,4,4,4-nonafluorobutane-1-sulfonate ([C4]) and bis­(trifluoromethylsulfonyl)­amide ([TFSI]). The synthesis procedures for the ionic liquids and the method for preparing the IL-Pt/C composites are detailed in ref 6. Specific purity information for the ionic liquids is not available from the original synthesis. For the systems with ionic liquid, the carbon components were impregnated with ionic liquid, as detailed in ref 6. TKK TEC10E50E was employed for Pt/C samples whereas Ketjenblack EC-300J was used for the Pt-free sample.^13^ The solutions were prepared for measurement by sequential mixing of the carbon component, water, ethanol, and D2020 (Chemours Nafion D2020 Dispersion), in this order. After the addition of the water and D2020, the sample was sonicated for 15 min in an ice bath. The carbon:water:ethanol:D2020 mass ratio was kept at 0.13:1:0.7:0.46 for all samples. To vary the neutron contrast of the samples, equal mass fractions of the water and ethanol were substituted for D_2_O and ethanol-*d*_6_, respectively. At least six contrast conditions were prepared for each ink system, with the deuterated content being varied between 0 and 100% deuterated.

**Figure 1:**
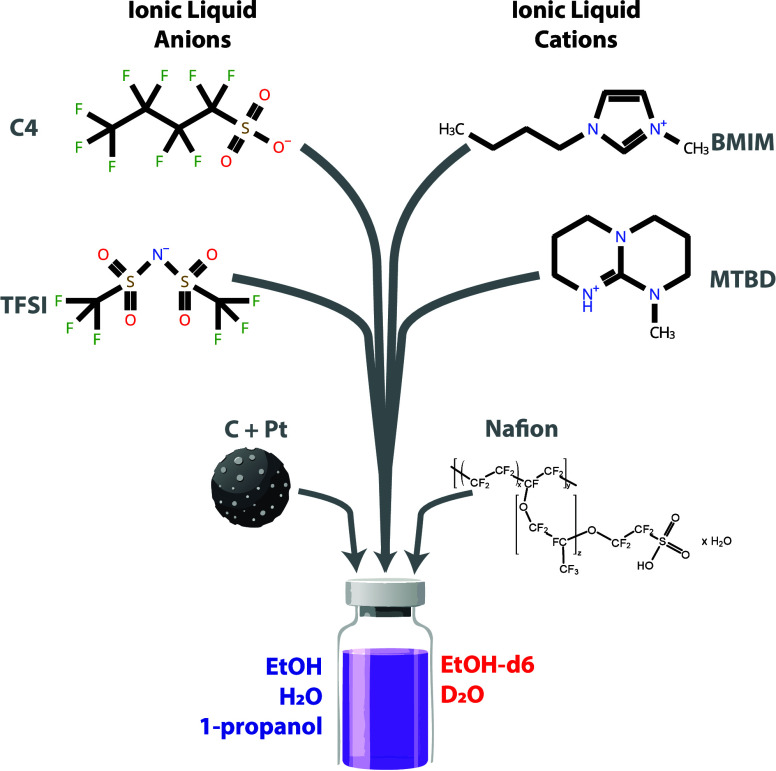
Schematic of an ionomer ink in this study with a neutron contrast variable suspending solvent.

### Small-Angle Neutron Scattering

SANS data were collected at the National Institute of Standards and Technology (NIST) Center for Neutron Research (NCNR) on the *n*Soft 10 m SANS instrument. Data were collected in three configurations at room temperature over two wavelengths (λ = 12 and 5 Å) and two sample-to-detector distances (1.15 and 4.65 m) to achieve a combined wavenumber range of $q=\frac{4\pi }{\lambda }\sin\left(\theta \right)$ ≈ 0.003 to 0.5 Å^–1^.

All SANS data were reduced and azimuthally integrated using the NCNR SANS Igor Macros.^14^ After reduction, the incoherent background of each 1D data set was subtracted using the following approach. First, the flat region at the high wavenumber end of each data set was identified by its first derivative, which was calculated using a Savitsky-Golay filter, as implemented in Scipy version 1.9.3 with parameter window_length = 9 and polyorder = 2, and deriv = 1.^15^ The smallest wavenumber, where the first derivative of the data was less than −0.1, *q*_min_^incoh^, was used as a heuristic to programmatically identify the portion of the data that was dominated by incoherent background. In this region, the slope of the linear fit to *q* ^4^ *I*(>*q*_min_^incoh^) vs *q* ^4^ is the incoherent background and is subtracted from the data set to yield the coherent scattering signal.

### Contrast-Variation SANS

CV-SANS has been described previously,^9,10^ so we will only summarize the approach here. As stated above, we decompose the SANS intensity measured at *n* contrast conditions, *I*_*n*_(*q*), into partial scattering functions, *S*_*ij*_(*q*), between all pairs of components *i* and *j* in a multicomponent mixture. In this study, we seek the partial scattering function for 3 components: the ionomer polymer (*N*), the ionic liquid (*I*), and the composite C + Pt support particle (*P*). Mathematically, the CV-SANS formalism is described as

1 $$\left[\begin{array}{c} I_{1}\left(q\right)\\ I_{2}\left(q\right)\\ I_{3}\left(q\right)\\ ...\\ I_{n}\left(q\right) \end{array}\right]=\left[\begin{array}{cccccc} \Delta \rho_{\mathrm{PP}}^{1}\left(q\right) & \Delta \rho_{\mathrm{PN}}^{1}\left(q\right) & \Delta \rho_{\mathrm{PI}}^{1}\left(q\right) & \Delta \rho_{\mathrm{NN}}^{1}\left(q\right) & \Delta \rho_{\mathrm{NI}}^{1}\left(q\right) & \Delta \rho_{\mathrm{II}}^{1}\left(q\right)\\ \Delta \rho_{\mathrm{PP}}^{2}\left(q\right) & \Delta \rho_{\mathrm{PN}}^{2}\left(q\right) & \Delta \rho_{\mathrm{PI}}^{2}\left(q\right) & \Delta \rho_{\mathrm{NN}}^{2}\left(q\right) & \Delta \rho_{\mathrm{NI}}^{2}\left(q\right) & \Delta \rho_{\mathrm{II}}^{2}\left(q\right)\\ \Delta \rho_{\mathrm{PP}}^{3}\left(q\right) & \Delta \rho_{\mathrm{PN}}^{3}\left(q\right) & \Delta \rho_{\mathrm{PI}}^{3}\left(q\right) & \Delta \rho_{\mathrm{NN}}^{3}\left(q\right) & \Delta \rho_{\mathrm{NI}}^{3}\left(q\right) & \Delta \rho_{\mathrm{II}}^{3}\left(q\right)\\ & & & ... & & \\ \Delta \rho_{\mathrm{PP}}^{n}\left(q\right) & \Delta \rho_{\mathrm{PN}}^{n}\left(q\right) & \Delta \rho_{\mathrm{PI}}^{n}\left(q\right) & \Delta \rho_{\mathrm{NN}}^{n}\left(q\right) & \Delta \rho_{\mathrm{NI}}^{n}\left(q\right) & \Delta \rho_{\mathrm{II}}^{n}\left(q\right) \end{array}\right]\times \left[\begin{array}{c} S_{\mathrm{PP}}\left(q\right)\\ S_{\mathrm{PN}}\left(q\right)\\ S_{\mathrm{PI}}\left(q\right)\\ S_{\mathrm{NN}}\left(q\right)\\ S_{\mathrm{NI}}\left(q\right)\\ S_{\mathrm{II}}\left(q\right) \end{array}\right]$$

Here, Δρ_*ij*_^*n*^ = (ρ_*i*_ – ρ_solvent_^*n*^) (ρ_*j*_ – ρ_solvent_^*n*^), with ρ_*i*_ being the SLD of component *i* and ρ_solvent_^*n*^ is the SLD of the effective medium solvent at contrast condition *n*. The SLDs used in this study are shown in Table 2. Equation 1 is solved by minimizing the following expression:

2 $$\delta =\frac{1}{n_{q}}\underset{q}{\sum }{\left(\frac{I\left(q\right)-\Delta \rho \cdot S\left(q\right)}{I\left(q\right)}\right)}^{2}$$

where *n*_*q*_ is the number of *q* in each measured *I* ^*n*^(*q*) and *I*(*q*), Δρ, and *S*(*q*) refer to the three matrices in eq 1 from left to right, respectively. This expression is minimized using the L-BFGS-B algorithm, as implemented in Scipy version 1.9.3.^15^

**Table 2:** Scattering Length Densities Used in This Study

species	density [g/cm^3^]	SLD [×10^–6^ Å^–2^]	source
ionomer		3.250	^9^,^10^
carbon	1.8	6.000	calcd
platinum	21.45	6.357	calcd
particle, Pt/C		6.025	calcd: 47% by mass Pt + C
IL1	1.4	2.028	calcd
IL2	1.4	2.058	calcd
IL3	1.4	1.927	calcd

## Results and Discussion

### Structural Analysis through Unified Fitting

Figure 2a shows the background subtracted SANS data for the six systems shown in Table 1 at two neutron contrast conditions. In Figure 2a, the measurements were conducted in hydrogenated solvents (see Sample Preparation), and we see little variation in the data between the various systems. For this system, the effective medium solvent has a SLD of ρ_solvent_ ≈ −0.45 × 10^–6^ Å^–2^. The carbon–platinum support particles have the largest contrast difference with Δρ_carbon,solvent_^2^ = (ρ_carbon_ – ρ_solvent_)^2^ ≈ 4.4 × 10^–11^ Å^–4^ and therefore dominate the signal. The data in Figure 2a suggest that variations in the particle composition, including the presence of and type of ionic liquid, do not strongly affect the arrangement of the particles. In this case where the carbon contrast is maximized, we observe that the carbon-only system (C, circles) and the platinum-containing carbon system (PtC, x symbols) exhibit nearly identical scattering profiles. This similarity is somewhat surprising because if platinum significantly affected the spatial arrangement or aggregation of the carbon support particles, we would expect to see measurable differences in their scattering patterns under these high-carbon-contrast conditions. The lack of such differences indicates that the presence of Pt does not substantially alter the structural organization of the carbon support network. Previous studies^16^ have suggested that the embedded platinum affects the particle aggregation structure through modulation of the particle–particle interactions. These data do not support this conclusion.

**Figure 2:**
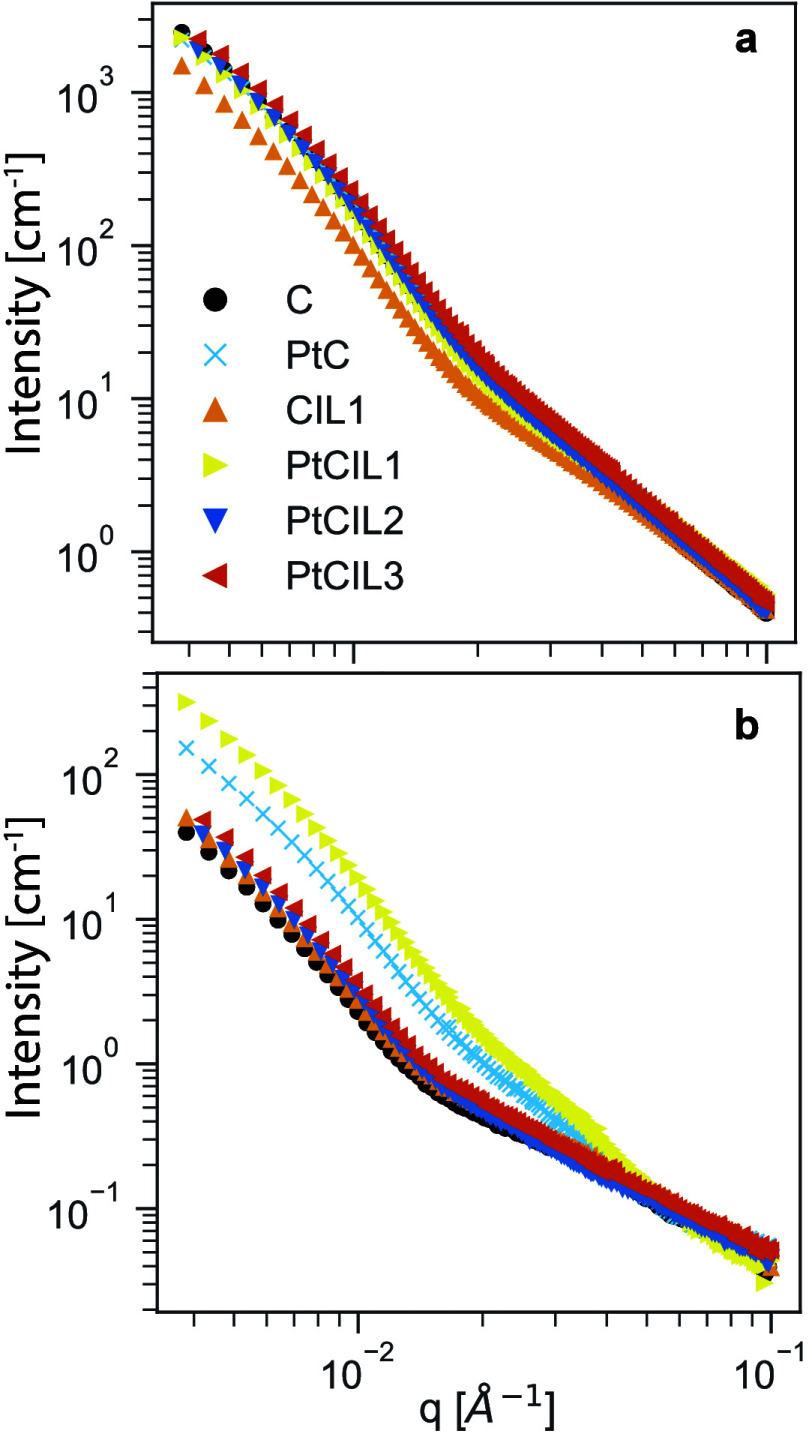
SANS data for the six systems described in Table 1 at two solvent contrast conditions with (a) ρ_solvent_ ≈ −0.45 × 10^–6^ Å^–2^ and (b) ρ_solvent_ ≈ 5 × 10^–6^ Å^–2^.

In Figure 2b, the measurements are conducted in a mixture of hydrogenated and deuterated solvents such that the effective solvent SLD is ρ_solvent_ ≈ 5 × 10^–6^ Å^–2^. This is the upper limit of contrast for the solvent without conducting a solvent-exchange process on the commercial ionomer suspensions. At these concentrations, the ionomer has the highest contrast (Δρ_ionomer,solvent_^2^ ≈ 2.84 × 10^–12^ Å^–4^), but the carbon is not perfectly contrast matched (Δρ_carbon,solvent_^2^ ≈ 1.55 × 10^–12^ Å^–4^) to the suspending solvent. Due to the strong scattering of the carbon particles, this means that carbon still contributes significantly to the scattering signal. Despite this, we observe more significant variation between the different systems in Figure 2b than Figure 2a. In particular, the PtCIL1 system ([BMIM]­[C4]) shows significantly increased scattering at all wavenumbers. At low- and mid-*q*, there is an increased power law slope, indicating a change in the long-range order of the composite.

To better understand these data, we first apply a traditional, empirical scattering model: the Unified Power Law Model, also known as the “unified scattering function” or simply the “unified model”.^17^

3 $$I\left(q\right)=\overset{N}{\underset{i}{\sum }}G_{i}\exp\left(-\frac{q^{2}R_{g,i}^{2}}{3}\right)+\exp\left(-\frac{q^{2}R_{g,i+1}^{2}}{3}\right)B_{i}{\left(q^{*}\right)}^{-P_{i}}$$

4 $$q^{*}=\frac{q}{\mathrm{erf}{\left(\frac{kqR_{g,i}}{\sqrt{6}}\right)}^{3}}$$

In the above expressions, *N* = 2 is the number of levels in the model, *R*_g,*i*_ is the radius of gyration of level *i*, *P*_*i*_ is the fractal dimension of level *i*, *G*_*i*_ is the Guinier scaling coefficient of level *i*, *B*_*i*_ is the Porod scaling coefficient of level *i*, and *k* = 1.06. We use the unified model as implemented in the Irena tool suite.^18^ This model has been used to describe the neutron and X-ray scattering of fractal systems, including polymers, nanoparticle assemblies and polymer/nanoparticle composites.^19–22^ We chose the unified model over other analytical approaches, such as specific form factor models (e.g., spheres, rods), because of its ability to describe hierarchical systems with multiple characteristic length scales. While specific form factor models assume well-defined geometric shapes, these ionomer solutions exhibit more complex morphologies that are better described by the unified model’s combination of Guinier and power-law regimes. Other studies of ionomer solutions have employed form-factor based approaches, such as using core–shell models for ionomer/particle aggregates.^11^ In our case, the simultaneous presence of ionomer chains, ionic liquid molecules, and carbon-supported catalyst particles creates a multicomponent system with overlapping size scales that cannot be adequately captured by any single form factor model.

Figure 3 shows three of the fitted unified parameters (*R*_g,1_, *R*_g,2_, and *P*_1_), while Figure S1 shows a representative fit to the data. Intuitively, the level 1 parameters are most likely descriptive of the ionomer chains. *R*_g,1_ represents the smaller characteristic length scale that appears in the scattering data. Based on the magnitude of this length scale and the known dimensions of our system components, we attribute this most likely to ionomer chain structures, though we acknowledge this assignment is somewhat indirect. It is important to note that this parameter does not specifically probe individual chain dimensions, but rather represents a characteristic size scale of the ionomer organization within the complex multicomponent system, which could include both single-chain conformations and small ionomer aggregates. *R*_g,1_ and *P*_1_ roughly mirror each other as one would expect. As the radius of gyration of the ionomer chains decreases the fractal dimensions of the chain increase, indicating more compact chain structures. For polymer chains, *P*_*i*_ describes a mass fractal related to the Flory chain dimension $\nu =\frac{1}{P_{i}}$ . A *P*_*i*_ = 2.0 would indicate ideal, random-walk chain conformations, while a *P*_*i*_ = 3.0 is indicative of chain collapse.

**Figure 3:**
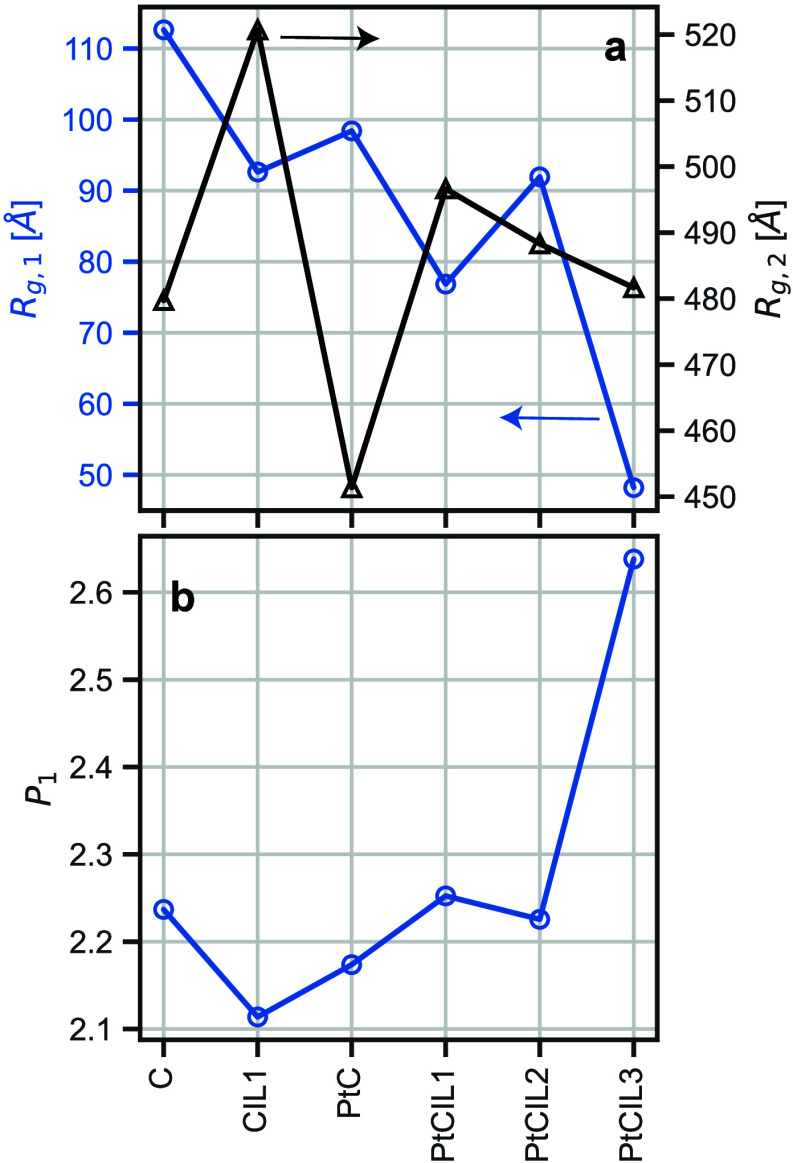
(a) Radius of gyration, *R*_g,*i*_, and (b) power law exponent, *P*_*i*_, fit parameters from the Unified Power Law model. Nearly identical fit-parameters and quality of fits were achieved for both contrast conditions shown in Figure 2.

The variation of these parameters across the three ILs suggests that the IL choice changes the conformation of the polymer chain, potentially through a variety of different mechanisms. Charge screening by the ILs could alter the electrostatic repulsion between sulfonate groups along the ionomer backbone, leading to more compact conformations in the presence of high ionic strength environments. Alternatively, competition for surface interactions with the Pt and C surfaces could modify the ionomer conformation by anchoring specific segments to the particle surfaces. The notably lower *R*_g_ and higher fractal dimension for IL3 ([MTBD]­[TFSI]) relative to IL1 and IL2 suggests that this IL combination may provide more effective charge screening or different surface interaction characteristics, though we emphasize that these are speculative explanations. Unfortunately, the unified model analysis contains contributions from all components simultaneously, making it challenging to definitively attribute specific structural changes to individual components without the higher-level CV-SANS analysis that follows.

*R*_g,2_ likely describes the primary carbon particles and its relative variation is much smaller compared to the *R*_g,1_. This easily follows from the relative compressibility of the carbon particles compared to the polymer chains. In order to reduce the number of fitting parameters and increase the robustness of the fit optimizer, the fractal dimension of the second level (not shown in Figure 3b) was fixed to a value of *P*_2_ = 4.0 for all systems. A value of *P*_2_ = 4.0 indicates a surface fractal describing an interface that is “smooth” relative to the length scale of the interface which, in this case, refers to the solvent–particle interface.

### Structural Analysis through CV-SANS

To further understand the effect of the ILs on the ink structure, we use the contrast variation SANS method (CV-SANS). In this approach, the scattering data is decomposed into contributions from and between the various components of the ink, i.e., the particles (*P*), the ionomer (*N*), and the ionic liquid (*I*). In our construction, the decomposed “particle” scattering includes both the carbon support and the platinum, while the IL terms include both the cation and the anion of the IL pair. The CV-SANS approach allows us to mathematically circumvent the chemical limitations of contrast matching and elucidate the structural contributions of the individual components.

Figure 4 shows the six scattering functions resulting from the decomposition of the data in Figure 2. To begin, we consider the “self-terms” (i.e., *S*_*ij*_(*q*), where *i* = *j*) in Figures 4a–c, which can be analyzed identically to nondecomposed SANS data. We see that the support scattering, *S*_*PP*_ in Figure 4a is mostly unchanged with varying ionic liquids or the presence/absence of Pt. This confirms our initial observations on the data in Figure 2a and is in line with our analysis of the unified fits discussed above: the ionic liquids are not strongly affecting the arrangement or aggregation of the support particles. In contrast, there are large changes to the scattering of the ionomer (Figure 4b). In particular, we see increasing scattering intensity at low-*q* going from the PtCIL2 to PtCIL3 to PtCIL1. We also note that, comparing the C system to all others in Figure 4b, the lack of signal at low-*q* indicates no large-scale structure formation. These data and the ionomer–particle *S*_*NP*_, data shown in Figure 4e and discussed below, suggest that the choice of IL and the presence of Pt strongly affects the organization of the ionomer.

**Figure 4:**
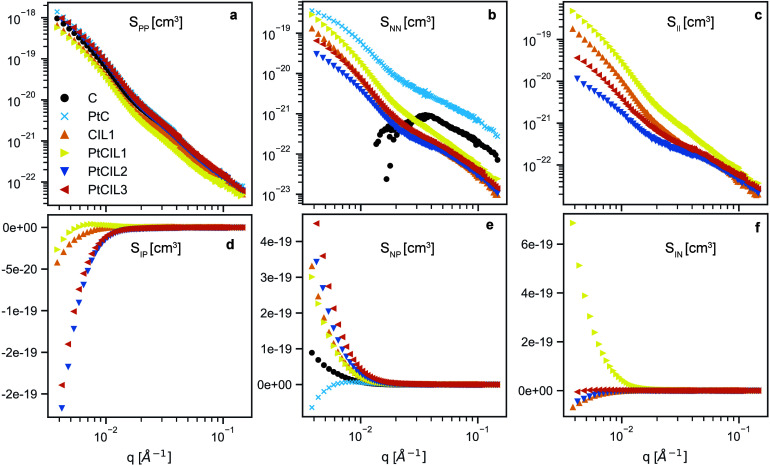
CV-SANS decomposition of SANS data into the contributions from (a) carbon/Pt (support) *S*_*PP*_, (b) ionomer, *S*_*NN*_, and (c) ionic liquid *S*_*II*_, along with the cross terms quantifying the interactions between the components: (d) ionomer–particle *S*_*NP*_, (e) “ionic liquid”–particle *S*_*IP*_, and (f) “ionic liquid”–ionomer *S*_*IN*_.

Guinier fits to the low-*q* region of the *S*_*NN*_ region indicate an increasing length scale of ≈322, 331, and 366 Å for PtCIL2, PtCIL3, and PtCIL1, respectively (Figure S2). Since these length scales are larger than the expected *R*_g_ of the ionomer, they are likely indicative of the ionomer’s arrangement around the support particle aggregates rather than the single-chain conformation of the ionomer itself. This is consistent with previous studies that describe ionomers adsorbing onto the catalyst support aggregates, although some studies also highlight the presence of excess, nonadsorbed ionomer in solution that deposits and binds particles during the drying process.^10,11^ We note that the *R*_g_ trends shown in Figure S2 do not match those shown in Figure 3 from the unified analysis. This discrepancy likely lies in the assignment of features in the nondecomposed scattering data to specific components when, in reality, multiple components can contribute to a scattering feature. The power of the CV-SANS approach is to provide an analysis with less ambiguity with respect to chemical identity.

The CV-SANS analysis also allows us to extract the contribution to the scattering from the ILs themselves. In Figure 4c, we show the IL scattering, *S*_*II*_ which qualitatively mimics the behavior of the ionomer at large-*q*. Given that both the ionomer and ILs are likely strongly interacting with the support particles, it follows that both of their long-range interactions are similar. The Guinier analysis shown in Figure S4 shows that the fitted length scales at low-*q* are similar as well.

Moving beyond the self-terms, we can analyze the cross interaction scattering functions between the particles, ionomer, and ILs (*S*_*NP*_, *S*_*IN*_, *S*_*IP*_, respectively). Unlike the self-scattering functions which only have physical meaning when they are positive, the sign of the cross term scattering is indicative of the type of interaction between those components.^10^ Positive scattering at low-*q* indicate that the two components are strongly associating while negative values indicate disassociation and weak interactions. A scattering value of zero at all *q* would indicate an ideal condition where the effective excluded volume of the two components become negligible, similar to the θ-conditions of polymer melts. While this qualitative cross-term analysis provides valuable qualitative information about component interactions, more quantitative interpretation is challenging for several reasons. Traditional analytical models commonly used to model SANS data cannot be applied to these cross terms. The inverted, real-space correlation function from a cross term, *p*_*i*,*j*_(*r*) describes the pair correlations between two different components in the system, but performing this inversion is numerically challenging and requires specific features to be present in the data.^23^ In our complex four-component system, the overlapping size scales and unknown interaction geometries make detailed quantitative modeling beyond the scope of the current analysis.

In Figure 4f we see that *S*_*IN*_ is only positive for PtCIL1 and is negative for all other ink systems. This suggests that the IL in PtCIL1 ([BMIM]­[C4]) are associating strongly with the ionomer while the other two IL pairs do not strongly interact with the ionomer. A potential explanation for these data is that the [BMIM] cation from IL1 interacts more favorably with the negatively charged sulfonate than the [MTBD] cation found on IL2 and IL3. Previous contact-angle measurements show that ILs with [BMIM] compared to [MTBD] cations had lower contact angles on Pt films.^7^ This supports the theory that Pt-[BMIM] interactions are more favorable than Pt-[MTBD] interactions. The origin of this phenomena behind this could be related to steric hindrances associated with the bulky [MTBD] fused ring compared to the single ring of [BMIM], or there could be a more subtle thermodynamic origin.

The trends seen in *S*_*IN*_ are also apparent in *S*_*IP*_ and *S*_*NP*_ (Figure 4d and e, respectively), although they are less pronounced. *S*_*IP*_ for PtCIL1 is less negative than PtCIL2 and PtCIL3 at low-*q* values, but is positive for most *q*. The larger, more positive values in *S*_*IN*_ compared to *S*_*IP*_ for PtCIL1 suggests that [BMIM]­[C4] preferentially associates with the ionomer over the Pt particles. Furthermore, the reduced *S*_*NP*_ at low *q* for PtCIL1 compared to PtCIL2 and PtCIL3 suggests reduced interactions between the ionomer and the catalyst particles. Taken together, these data suggest that PtCIL1 drives increased IL–ionomer and ionomer–ionomer interactions at the expense of some ionomer–particle interactions. This configuration is schematically depicted in Figure 5.

**Figure 5:**
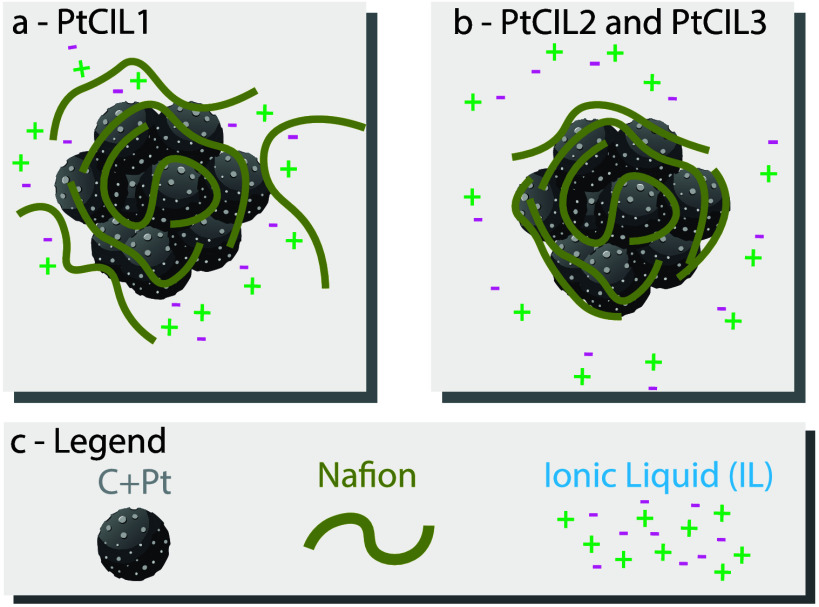
Schematic comparing the ink structure of (a) PtCIL1 and (b) PtCIL2 and PtCIL3 based on the interpretation of the CV-SANS results in Figure 4. Part c is a legend that identifies the graphics shown in parts a and b.

Interestingly, we can also note the effect of the Pt by comparing CIL1 to PtCIL1 across the decomposed scattering functions. *S*_*NN*_ in Figure 4b shows that the scattering intensity at low-*q* shows decreased intensity in the platinum-free case (CIL1) indicating that the presence of Pt modifies the ionomer structure. Analyzing the cross terms, we see that the two inks show similar scattering for *S*_*NP*_, while *S*_*IP*_ has positive regions only for the Pt case. This indicates that the ionomer–particle interactions are mostly unchanged by Pt, while “ionic liquid”–particle interactions are strengthened to some degree. In contrast, as shown in shown in Figure 4f, we observe stark changes to *S*_*IN*_ between the CIL1 and PtCIL1 systems, indicating greatly increased interactions between the ionomer and ionic liquid when Pt is present. Combining the observations from these three partial scattering functions suggests that, rather than simple competition for binding sites, the Pt creates a more complex three-component interaction network where the IL preferentially organizes around ionomer chains that are themselves interacting with the Pt particles.

### Connecting Structure to Performance

While the analysis of the previous section served to elucidate how the ILs were affecting the organization of the inks, our true purpose is to understand how this structure is linked to the final material performance in PEMFCs. Previous work has shown that ILs do modify the oxygen reduction reaction (ORR) activity but we seek to clarify whether this change is entirely due to electrochemical differences between the ILs or whether there is a structural origin.^5–7^

Figure 6 quantifies the performance of MEAs made from ink formulations with ILs. These data were previously reported in refs 6 and 7 and full experimental details for these data can be found there. Our work deviates from these references by substituting a fraction of the hydrogenated suspending solvent components (H_2_O and ethanol) with their deuterated analogues (D_2_O and ethanol-*d*_6_); This isotopic change should not affect the structure of the ink. We observe that PtCIL1 has lower activities (MA and SA) and fewer accessible Pt sites (1−θ_OH_) than either PtCIL2 or PtCIL3. Compared to PtCIL2 and PtCIL3, PtCIL1 was a clear outlier in the structural analysis, showing much stronger interaction with the ionomer and particles as shown by *S*_*IN*_ and *S*_*IP*_, respectively. The 1−θ_OH_ data, combined with our CV-SANS results, suggest that IL1 creates a complex interaction network involving both ionomer and Pt components. Rather than simple site blocking, our CV-SANS data shows that [BMIM]­[C4] exhibits strong interactions with both components, with a preference for ionomer association as evidenced by the larger magnitude and positive values of *S*_*IN*_ compared to *S*_*IP*_. The reduced accessible Pt sites likely result from this preferential IL-ionomer association creating a more organized but less accessible interfacial structure around the Pt particles.

**Figure 6:**
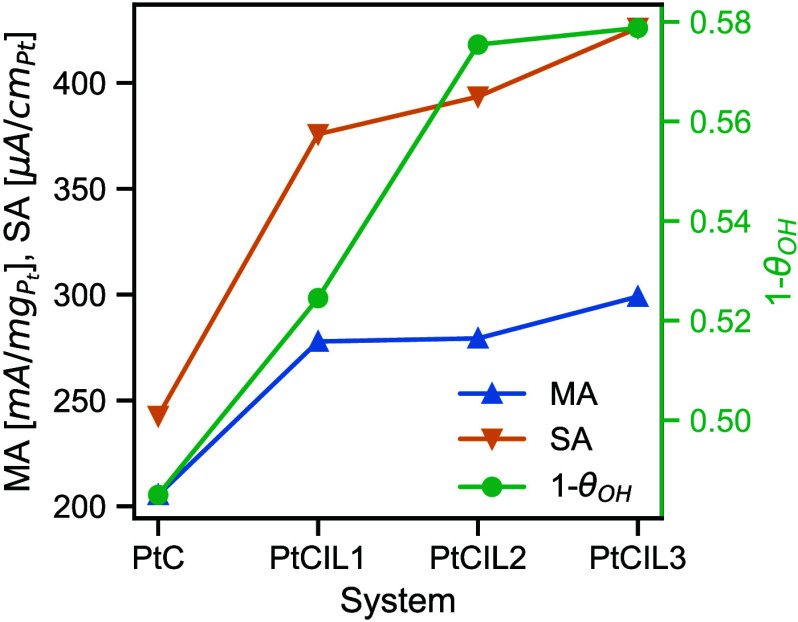
Mass activity (MA), specific activity (SA), and accessible Pt sites (1−θ_OH_) of MEAs created from four of the systems considered in this study. These data were previously reported in refs 6 and 7.

Overall, the CV-SANS analysis and the performance data align to show that the choice of IL pair affects the interactions and liquid structure of the ink constituents. This liquid structure serves as the template during film casting and therefore directly affects the electrochemical activity of devices made from the inks.^10,12^

## Conclusions

We have studied the ink-precursors of the catalyst layers of PEMFCs with the goal of correlating the ink microstructure with the performance of PEMFCs made from these inks. Specifically, we sought to correlate how the inclusion of ionic liquids affected structure-performance correlations. First, using a traditional analytical model, we fit the scattering data and quantified the length scales and fractal dimensions as a function of ink composition. Due to the SLDs of the ink components, attributing these features to specific chemical interactions (i.e., between Pt/C, ionomer, or IL) was not possible. Using the CV-SANS method, we were able to show that the choice of IL pair does not affect the arrangement of the Pt/C support particles but does change the conformations and arrangement of the ionomer. One of the IL pairs, IL1 = [BMIM]­[C4], shows stronger interactions with the ionomer and Pt-Carbon support particles compared to the other two ILs considered. The performance data aligns with the structural analysis showing that IL1 shows reduced activity and free Pt sites compared to IL2 and IL3. In particular, the reduced Pt site data supports CV-SANS conclusion that IL1 interacts strongly with the particles, thereby blocking free Pt sites.

Overall, this work shows that, by using more advanced analyses, SANS measurements can be used to elucidate subtle changes to structural arrangement in multicomponent systems. Furthermore, the understanding that the [BMIM]­[C4] ionic liquid leads to reduced ionomer–Pt/C interaction and thereby reduced PEMFC performance will motivate researchers to design better ILs that lead to better performance.

## Supplementary Material



## Notes

Example of the unified model fit to SANS data, and low-*q* Guinier analysis of decomposed scattering functions for all component pairs (PDF)
