# Supplementary material for: Effect of Ionic Liquids on the Structure of Ionomer Inks
Source: Macromolecules. 2025 Jul 28;58(15):8288–94. doi: 10.1021/acs.macromol.5c00698 (PMC12356067; doi:10.1021/acs.macromol.5c00698)
Supplement: Supplementary file 1 [file ma5c00698_si_001.pdf]

Supporting Information for "Effect of Ionic Liquids on the Structure of Ionomer Inks"

Tyler B. Martin*,† and Kimber Stamm Masias*,‡

†*Materials Science and Engineering Division, National Institute of Standards and Technology, Gaithersburg, Maryland, 20899, USA*

‡*Toyota Motor North America R&D, Ann Arbor, MI, 48105, USA*

E-mail: tyler.martin@nist.gov; kimber.stamm@toyota.com

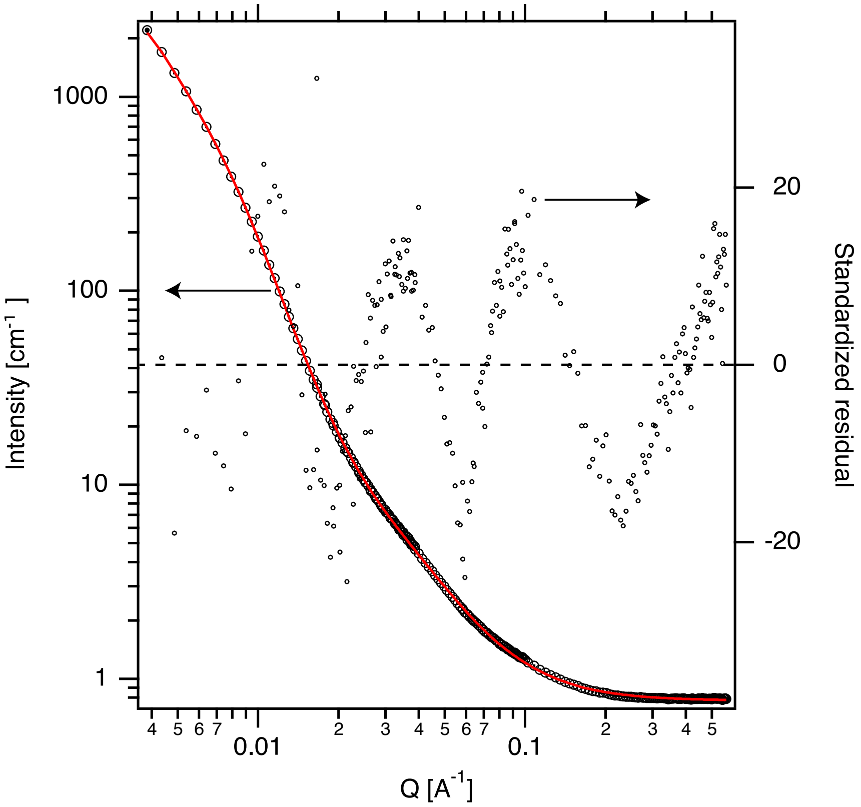

Figure S1: Example of fit to experimental data using the Unified Power Law Model for sample PtCIL1. The figure shows the scattered intensity [1/cm] versus wavenumber q [1/A] (open circles) with the unified model fit (solid red line). The figure also shows the standardized residuals (small black points) representing the difference between experimental and fitted values, normalized by the experimental uncertainty.

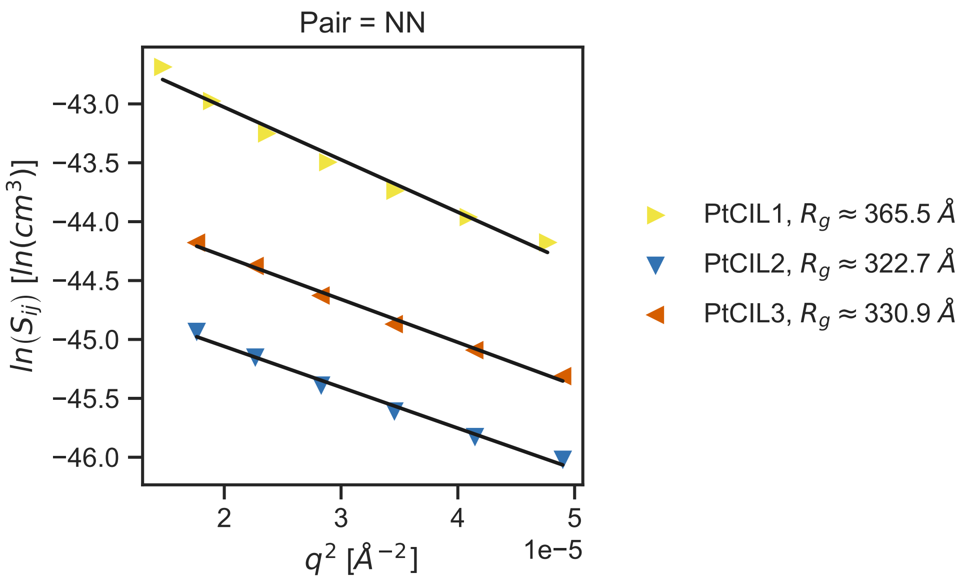

Figure S2: Low-q Guinier fits for CVSANS decomposed ionomer-ionomer scattering.

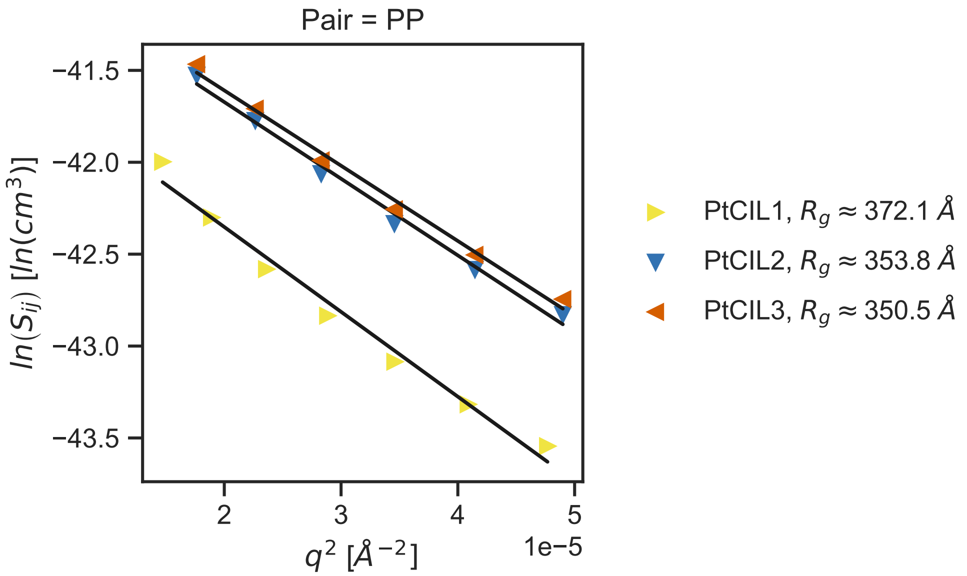

Figure S3: Low-q Guinier fits for CVSANS decomposed Particle-Particle scattering.

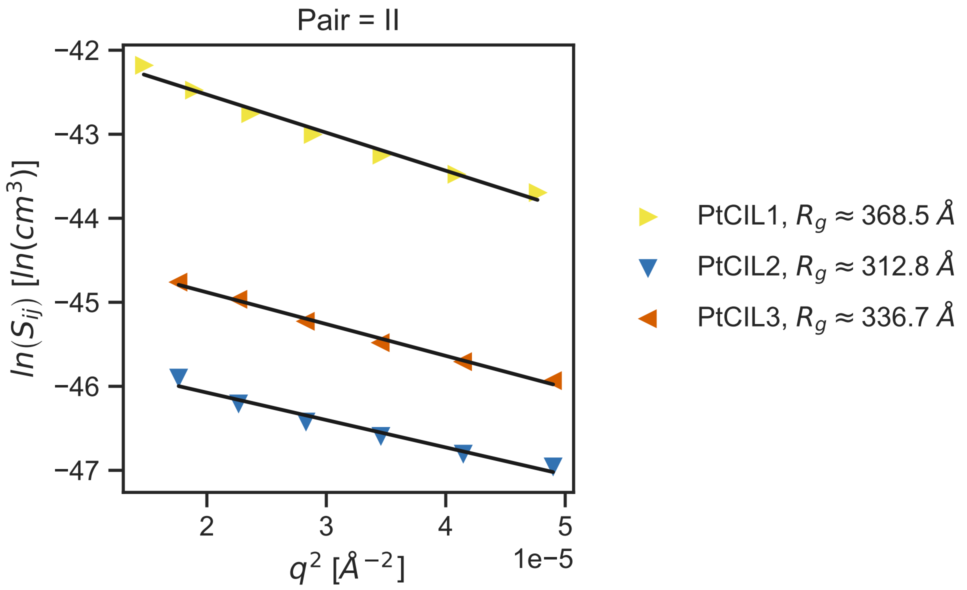

Figure S4: Low-q Guinier fits for CVSANS decomposed Ionic Liquid - Ionic Liquid scattering.
